# Enhancing Psychotherapy Outcomes by Encouraging Patients to Regularly Self-Monitor, Reflect on, and Share Their Affective Responses Toward Their Therapist: Protocol for a Randomized Controlled Trial

**DOI:** 10.2196/55369

**Published:** 2024-04-08

**Authors:** Alberto Stefana, Eduard Vieta, Paolo Fusar-Poli, Eric A Youngstrom

**Affiliations:** 1 Department of Brain and Behavioral Sciences University of Pavia Pavia Italy; 2 Department of Psychology and Neuroscience University of North Carolina at Chapel Hill Chapel Hill, NC United States; 3 Bipolar and Depressive Disorders Unit, Hospital Clinic, IDIBAPS, CIBERSAM, University of Barcelona, Barcelona Spain; 4 OASIS Service, South London and Maudsley NHS Foundation Trust London United Kingdom; 5 Early Psychosis: Interventions and Clinical-detection Lab, Department of Psychosis Studies, Institute of Psychiatry, Psychology & Neuroscience King’s College London London United Kingdom; 6 Institute for Mental and Behavioral Health Research, Nationwide Children’s Hospital, Division of Child and Family Psychiatry, The Ohio State University Columbus, OH United States; 7 Helping Give Away Psychological Science Chapel Hill, NC United States; 8 Department of Psychiatry The Ohio State University Columbus, OH United States

**Keywords:** adult patients, evidence-based assessment, patient’s perspective, psychotherapy, randomized controlled trial, systematic client feedback, therapeutic relationship, treatment outcome

## Abstract

**Background:**

The quality of the therapeutic relationship is pivotal in determining psychotherapy outcomes. However, facilitating patients’ self-awareness, reflection on, and sharing of their affective responses toward their therapist remains underexplored as a potential tool for enhancing this relationship and subsequent treatment outcomes.

**Objective:**

The primary objective of this study is to examine whether and how the patients’ regular self-monitoring and self-reflection (fostered by the systematic compilation of a brief postsession battery) on their affective reactions toward the psychotherapist impact the quality of the therapeutic relationship and treatment outcomes in individual psychotherapy. Secondary objectives are to (1) explore whether and how the characteristics of the patient, the therapist, and the process moderate the effect of regular self-monitoring on the therapeutic relationship and outcomes; (2) examine the relationships between the affective response of the patient, the alliance, and the result of the therapy session outcome; and (3) explore how the affective responses of the patient unfold or change throughout the course of the therapy.

**Methods:**

We conducted a 1:1 randomized controlled trial of adults in individual psychotherapy versus individual psychotherapy plus self-monitoring. Participants will be enrolled through the web-based recruitment platforms “ResearchMatch” and “Research for Me,” and data will be collected through web-based surveys. Participants in the control group will receive only their regular individual psychotherapy (treatment as usual) and will not complete postsession questionnaires. Participants in the intervention group will continue their regular individual psychotherapy sessions and complete the “in-Session Patient Affective Reactions Questionnaire” and the “Rift In-Session Questionnaire” following each therapy session in the 10 weeks of the trial. Additionally, after completion of the postsession battery, they will receive general written feedback encouraging them to discuss their feelings and reflections with their therapist. Participants in both groups will complete a comprehensive psychological assessment at baseline, midtrial (week 5), and end-of-trial (week 10). The primary outcome measure of the trial is the “Clinical Outcomes in Routine Evaluation-Outcome Measure,” while the secondary outcomes are the “Real Relationship Inventory-Client-Short Form,” the “Working Alliance Inventory-Short Revised,” and the number of scheduled therapy sessions that the patient has missed or canceled.

**Results:**

The trial was approved by the institutional review board of the University of North Carolina at Chapel Hill. Recruitment started in September 2023. A total of 475 individuals completed the baseline assessment. Data collection was completed in February 2024. The results are expected to be published in the autumn of 2024.

**Conclusions:**

This study could reveal key information on how regular self-monitoring and introspection can influence both the therapeutic relationship and treatment outcomes. Findings have the potential to shape interventions, enhance the efficacy of psychotherapeutic sessions, and possibly offer a cost-effective strategy for improving patients’ well-being.

**Trial Registration:**

ClinicalTrials.gov NCT06038747; https://classic.clinicaltrials.gov/ct2/show/NCT06038747

**International Registered Report Identifier (IRRID):**

DERR1-10.2196/55369

## Introduction

### Background

Emotions lie at the core of human existence, emerging as products of evolutionary mechanisms designed to equip individuals to handle critical interpersonal interactions and life challenges [1,2]. Since the dawn of psychotherapy, the role of emotions, whether emanating from the patient or the therapist, has been recognized as crucial to the therapeutic journey [3-5].

Studies on emotional expression within the therapeutic realm emphasize their substantial influence on the outcomes of both single sessions and the entire treatment journey [2,6,7]. Emotions intertwine with both specific and generic therapeutic methodologies that underpin clinical success [8,9]. Facilitating patients to gain emotional awareness and use these emotions constructively stands central to therapeutic transformation [10,11]. For this transformative effect, therapists must engage collaboratively with the patient’s in-session emotions, guiding them through recognition, acceptance, regulation, and management [12-14].

The therapeutic setting can illuminate suppressed emotions, allowing patients to confront neglected self-awareness and experience new emotional dimensions. Such insights present valuable data on patients’ inherent needs and reactions, offering a platform to confront past fears and integrate new experiences [11,15]. Emotions directed toward the therapist during sessions hold significant implications for the therapeutic journey’s success [16,17]. An environment characterized by empathy and affirmation determines if the emotional engagement will steer the therapy positively or detrimentally [18]. Furthermore, such an environment is both the catalyst for change and the foundation for effective psychotherapy [19].

The dawn of the new millennium saw the advent of routine outcome monitoring and feedback [20]. These systems, which track and report the patient’s therapeutic progress, have seen widespread adoption and study in varied mental health contexts over recent years. These feedback mechanisms, unified by their use of standardized measures, monitor outcomes like well-being, symptomatology, and functional aspects, sometimes focusing on specific therapeutic processes like the working alliance [21]. Meta-analyses show that systematic feedback enhances therapy outcomes by reducing symptom intensity and attrition rates [22,23]. They also highlight reductions in treatment time frames and associated costs [24-26].

Given these insights, the American Psychological Association has been advocating for feedback and outcome monitoring since the early 2000s [27,28]. Feedback systems are now integral to mental health frameworks globally, especially in countries such as Australia, the Netherlands, and the United Kingdom, where monitoring outcomes is a statutory mandate for health service providers. With the capacity to tweak treatments based on progress or disruptions in the therapist-patient relationship, many experts regard progress monitoring as a pivotal strategy to enhance real-world therapy outcomes [29,30].

### Study Objectives

In light of the above, the primary objective of this study is to examine the influence of a brief postsession battery, which is apt to foster patient self-monitoring and reflection on their emotional reactions toward their psychotherapist and on the quality of the therapeutic relationship and treatment outcomes.

Secondary objectives are to (1) explore whether and how the characteristics of the patient, the therapist, and the process moderate the effect of regular self-monitoring on the therapeutic relationship and outcomes; (2) examine the relationships between the affective response of the patient, the alliance, and the result of the therapy session outcome; and (3) explore how the affective responses of the patient unfold or change throughout the course of therapy.

## Methods

### Ethical Considerations

The institutional review board of the University of North Carolina (UNC) at Chapel Hill approved this study (23-1067) on July 31, 2023. This research was designed and executed in accordance with ethical standards for studies involving human participants.

Before starting the baseline survey, participants will receive a detailed web-based consent form regarding the study. This form will include the purpose, rationale, and methodology of the study, as well as the contact information for both the principal investigator and the institutional review board. It will be explained to potential participants that to protect their identities, only relevant and minimally necessary information will be collected. Furthermore, it will be assured that any published results will report on groups, not individual participants. Participants will be prompted with a consent statement and given options: “I consent to participate in this study” or “I do not consent to participate in this study.” Those who choose not to consent will be immediately directed to a closing page. All participants are informed that they can opt out of the study at any time without providing a reason.

Only the central research team will have access to the data until the research results are published. Once the study concludes and the primary findings are released, external applications for access to deidentified data will be entertained. These applications must adhere to data privacy rules, and the intended research must be scientifically and ethically robust. Following the publication of the research findings, the data will be publicly shared through the Open Science Framework [31].

No financial incentives were offered for participation in this trial. However, upon completing the trial, participants from both the intervention and control groups were given the opportunity to request a free copy of the scales and general feedback used in the intervention group by emailing the principal investigator.

### Study Design

This study is a randomized controlled trial (NCT06038747) of 1:1 for individual psychotherapy versus individual psychotherapy plus self-monitoring. A predetermined computer-generated randomization list will be generated to assign patients to 1 of the 2 arms. Patients will not be blind to treatment allocation. Patients will be enrolled through the web-based recruitment platforms “ResearchMatch” and “Research for Me,” and data will be collected through web-based surveys facilitated by the Qualtrics software hosted by the UNC at Chapel Hill.

### Intervention

Participants in the intervention group will continue their regular individual psychotherapy sessions. Additionally, after each session, they will complete a brief postsession battery consisting of 2 scales on the affective reactions of the patients toward their therapist during the session and read general feedback encouraging them to discuss their feelings and reflections with their therapist. This questionnaire aims to prompt reflection on one’s own experience of the therapeutic relationship.

### Treatment as Usual

Patients in the control group will receive only their regular individual psychotherapy (treatment as usual) and will not complete postsession questionnaires. Additionally, they will not receive any communication encouraging them to discuss their emotional responses with their therapist during the session.

### Measures

This study uses a comprehensive set of self-report instruments to collect extensive data on the therapeutic process and various patient-related factors. The data will cover 5 main domains. The sociodemographic and clinical domains capture the patient’s demographic, clinical, and treatment specifics. The mental health state domain focuses on the present symptoms of anxiety and depression that impact the daily lives of respondents. The therapeutic relationship domain evaluates the specific facets of the relationship between the patient and the therapist. The last domain evaluates session and therapy outcomes.

The primary outcome measure of the trial is the “Clinical Outcomes in Routine Evaluation-Outcome Measure” (CORE-OM) [32], while the secondary outcomes are the “Real Relationship Inventory-Client-Short Form” (RRI-C-SF) [33], the “Working Alliance Inventory-Short Revised” (WAI-SR) [34], and the number of scheduled therapy sessions that have been missed or canceled by the patient.

#### Sociodemographic Domain

The “sociodemographic data form” collects self-reported details such as the patient’s age, gender, education level, and ethnicity.

#### Clinical Domain

The “clinical data form A” gathers patient-provided data on the duration and frequency of the ongoing psychotherapy, session attendance method (in-person, video call, telephone, and mixed), presence or absence of a diagnosed mental disorder, any prescribed psychotropic medications, and the therapist’s gender.

The “clinical data form B” records information on sessions attended, missed, and canceled over the last 5 weeks and any change in psychiatric medications during this time frame.

#### Mental Health State Domain

The Patient Health Questionnaire-9 (PHQ-9) [35] is a 9-item self-report depression screening tool that captures the severity of depressive symptoms during the past week. It allows the evaluation of symptoms according to the Diagnostic and Statistical Manual of Mental Disorders, Fifth Edition (DSM-5) criteria for major depression. The PHQ-9 administered in the primary care sample showed a Cronbach α of 0.89.

The Generalized Anxiety Disorder-7 (GAD-7) [36] is a 7-item self-report anxiety screening tool with a focus on symptoms of generalized anxiety disorder. It assesses the severity and frequency of anxiety symptoms during the past week. GAD-7 showed a Cronbach α of 0.92.

#### Therapeutic Relationship Domain

The RRI-C-SF [33] is an 8-item self-report tool measuring the “real relationship” between a therapist and patient from the patient’s viewpoint. It includes 2 subscales: “genuineness” and “realism,” both of which represent closely related dimensions of authenticity and perceptual accuracy within the therapeutic relationship. The RRI-C-SF has a McDonald’s ω total of 0.93.

The WAI-SR [34] is a 12-item self-report measure of the quality of the working alliance between patient and therapist from the patient’s perspective. WAI-SR includes three 4-item subscales that focus on agreement on therapy tasks, goals, and the development of an emotional bond. Items are rated on a 6-point Likert scale, with higher scores indicating a more robust alliance. The Cronbach α for the total scale was 0.91, while the α coefficients for the subscales spanned a range from 0.85 to 0.90.

The “in-Session Patient Affective Reactions Questionnaire” (SPARQ) [37,38] is a patient-reported tool that comprises 8 items that explore the patterns of thought, feeling, and behavior activated and experienced by the patients toward their therapist during a session. It consists of 2 scales: “positive affect” and “negative affect.” The positive affect scale exhibited a Cronbach α coefficient of 0.84 and an average interitem correlation of 0.51. On the other hand, the negative affect scale demonstrated an α of 0.76 and an average interitem correlation of 0.39.

The “Rift In-Session Questionnaire” (RISQ) [37] is a 4-item self-report questionnaire designed to measure the patient’s risk of experiencing ruptures in the therapeutic relationship. The RISQ measures feelings of belittlement, rejection, disparagement, and attack, as well as any tendencies toward disobedience or “naughtiness” toward the therapist. The RISQ demonstrated good internal consistency with a Cronbach α coefficient of 0.73 and an average interitem correlation of 0.35.

#### Session and Therapy Outcomes Domain

The Session Evaluation Scale (SES) [39] assesses the quality of the therapy session from the perspective of the patient. The SES comprises 5 items rated on a 5-point Likert scale and showed Cronbach α coefficients ranging from 0.88 to 0.89.

The CORE-OM [32] is a 34-item self-report measure of change in psychotherapy that comprises 4 domains: subjective well-being, symptoms, function, and risk. The CORE-OM demonstrated good internal and test-retest reliability between 0.75 and 0.95, and convergent validity against a battery of validated measures.

### Efforts to Minimize Potential Sources of Bias

In our research, extensive steps will be taken to minimize potential biases. First, participants will be sourced from ResearchMatch, a national web-based registry that boasts a broad and varied volunteer pool from all over the United States, reducing selection bias and enhancing the external validity of our study. Second, our methodology is closely tuned to gender perspectives, giving weight to nonbinary gender as a key factor in both data evaluation and result interpretation, fortifying the scientific rigor and clinical significance of the study. Third, our comprehensive set of measures covers a spectrum of therapeutic and individual aspects, from sociodemographic details to personality characteristics, providing a comprehensive snapshot of the therapeutic landscape and limiting measurement bias. Data will be gathered through Qualtrics, a trusted web-based tool that protects respondent privacy and minimizes potential biases in responses. Furthermore, great care was taken in calculating the required sample size, accounting for potential dropouts, to ensure that we had ample statistical power, thereby sidestepping the pitfalls of weak analyses or insufficient representation.

### Sample Size Calculation

Assuming a power of 0.80, a 2-tailed α level of .05, an effect size (*d*) of 0.50, and an allocation ratio of 1:1, we calculated a minimum sample size of 128 patients, or 64 participants per group, using the *R package pwr* (version 1.3-0; The Comprehensive R Archive Network). However, to enhance statistical power and precision, we aim for a target total sample size of 520 participants, with 260 participants per group. This adjustment also accounts for the possibility that only 50% of participants in the intervention group will complete all postsession scales.

### Inclusion and Exclusion Criteria

Participants are eligible for the study if they are aged 18 years or older, fluent in English, and currently undergoing individual psychotherapy with a minimum frequency of 2 sessions per month. Individuals with a legal guardian will be excluded from the study.

### Recruitment

Patients will be enrolled through ResearchMatch and Research for Me. ResearchMatch [40] is a disease-neutral, institution-neutral, US national web-based registry designed to enroll volunteers for clinical studies. Established by various educational entities and supported in part by the National Institutes of Health’s National Center for Advancing Translational Sciences, this platform provides access to more than 155,000 volunteers across the United States and has proven effective. Research for Me is a community of volunteers acting as the primary gateway for patients and local residents who wish to participate in research at the UNC at Chapel Hill. This initiative was established by the North Carolina Translational and Clinical Sciences Institute, which is the central component of the National Institutes of Health’s Clinical and Translational Science Awards program at UNC at Chapel Hill. Data suggest that individuals enrolled through online research platforms reliably report their demographic and psychological details, especially when there is no monetary incentive involved [41].

### Randomization

Participants will be assigned to either the intervention or control group using a pre-established computer-generated randomization list. This assignment will occur after the baseline assessment is completed.

### Data Collection Process

Participants in both the intervention and control groups will undergo assessments at baseline, midtrial (week 5), and end-of-trial (week 10). Additionally, those in the intervention group will have postsession evaluations. These assessments will be delivered through Qualtrics, a secure web-based survey platform hosted by the UNC at Chapel Hill.

Baseline assessment: All participants will complete the demographic and clinical data forms in addition to the measures for mental health state, therapeutic relationship, and session and therapy outcome domains (Table 1).

**Table 1:** Instruments and time line for assessment in the trial.

Domains and measures		Baseline	Midtrial^a^	End-of-trial^b^	
**Demographic domain**					
	Sociodemographic data form	✓			
**Clinical domain**					
	Clinical data form A	✓			
	Clinical data form B		✓	✓	
**Mental health state domain**					
	Patient Health Questionnaire-9 [35]	✓	✓	✓	
	Generalized Anxiety Disorder-7 [36]	✓	✓	✓	
**Therapeutic relationship domain**					
	Real Relationship Inventory-Client-Short Form [33]	✓	✓	✓	
	Working Alliance Inventory-Short Revised [34]	✓	✓	✓	
	in-Session Patient Affective Reactions Questionnaire [37,38]	✓	✓	✓	
	Rift In-Session Questionnaire [37]	✓	✓	✓	
**Session and therapy outcome domain**					
	Session Evaluation Scale [39]	✓	✓	✓	
	Clinical Outcomes in Routine Evaluation-Outcome Measures [32]	✓	✓	✓	

^a^5 weeks after the baseline assessment.

^b^10 weeks after the baseline assessment.

Postsession assessment (only for the intervention group): participants in the intervention group will be required to complete SPARQ and RISQ [37] after each session throughout the 10-week duration of the trial. Immediately after completion of the postsession battery, participants will be presented with a general feedback statement emphasizing the importance of discussing session-related feelings with their therapist.

Mid-trial assessment: 5 weeks postbaseline evaluation, all participants will retake the scales used in the baseline assessment (Table 1).

End-of-trial assessment: 10 weeks after the baseline, all participants will again be asked to complete the initial assessment battery (Table 1).

### Dealing With Missing Values

We will examine data for degree and patterns of missingness at the patient and scale level, use propensity scoring to model the pattern of missingness, and include the propensity score as a covariate in analyses if indicated [42]. Missing items will be prorated so long as at least 75% of the items are available, and otherwise the scale score will be treated as missing [43].

### Planned Analysis

A comprehensive statistical strategy will be formulated and documented before starting the data analysis. R (R Foundation for Statistical Computing) will be used for data cleaning, labeling, scale scoring, and subsequent analyses. The basic characteristics of the study participants will be clearly described for each arm while also being aggregated to a sufficient level to reduce the risk of deductive identification. Baseline categorical variables will undergo a comparative evaluation between the intervention and control groups using chi-square evaluations. Continuous variables will be compared using 2-tailed *t* tests and Mann-Whitney *U* tests. Outcome metrics will be presented for each research group at various intervals. These outcomes will be detailed based on cumulative scores and the percentage of participants that demonstrate improvement from the start point.

### Statistical Analysis

Analysis of covariance will be used to evaluate the influence of treatment conditions on primary and secondary outcomes since it is the most appropriate statistical method for the analysis of continuous outcomes in RCTs [44,45]. Analysis of covariance is a linear regression in which treatment assignment and baseline scores are included as covariates. Patient clinical and demographic characteristics, as well as therapist demographic and professional characteristics, will be included as exploratory variables. A total of 2 analyses will be carried out: the intention-to-treat analysis to examine the general effect and the per protocol analysis as a sensitivity analysis. The time will be measured in weeks, and the baseline is coded with the value 0. Analyses will be performed using R statistical computing software (version 4.3.1 or higher; R Foundation for Statistical Computing).

### Trial Status

At the time of submission of this protocol’s manuscript, recruitment is complete.

### Dissemination Policy

The results of this research project will first appear as preprints and subsequently be shared through scholarly journals and presentations at conferences. The Open Science Framework will host a repository containing study tools and data, scoring guidelines, presentations, and preprint versions. Wikiversity pages, tailored to offer technical assets for both practitioners and researchers, will feature links directing to this repository. Furthermore, this study’s conclusions could be communicated to pertinent mental health associations, contributing to future studies and potential improvements in psychotherapeutic relationships. Our goal is to adopt a comprehensive and broad-based communication approach that engages scholars, health care professionals, and the general public.

## Results

Participant recruitment started in September 2023. Baseline data collection was completed in December 2023, with a total of 520 recruited participants, 475 of whom completed the baseline assessment (243 participants were assigned to the intervention group and 232 were assigned to the control group). Data was completed February 2024. Data analysis has not begun as of the time of submission. The results are expected to be published in the autumn of 2024.

## Discussion

This clinical study is designed to assess the impact of introducing a concise postsession questionnaire able to trigger the self-awareness and introspection of patients about their experience of the psychotherapeutic relationship. The primary goal is to understand its influence on the effectiveness of treatment, with the CORE-OM serving as the primary outcome measure.

Should our battery prove to increase symptom reduction rates or improve overall well-being, its ease of delivery directly to patients—requiring no additional therapist involvement—suggests potential for seamless adoption across therapeutic contexts without incurring extra expenses for patients or placing added demands on therapists. This could make the system highly cost-efficient. Furthermore, the insights from this study could shed light on the nuanced interaction between specific elements of the therapeutic bond and the results of sessions or general treatment. This knowledge would empower clinicians and policy makers to recognize and prioritize those aspects of the therapeutic relationship that strengthen or hinder the efficacy of psychotherapy.

One of the main strengths of this study is its ability to gauge the benefits of regular patient self-monitoring and subsequent reflection on their emotional reactions toward their therapists in a real-world setting. The study sample accurately represents the diverse patient population within the United States, further enhancing its relevance. Other key advantages include robust statistical power, the use of an external outcome measure not related to the intervention, and a comprehensive consideration of the patient, the therapist, and treatment variables. However, there are limitations, such as the lack of information from the clinician or observer and the extended duration of data collection. Furthermore, an important potential limitation inherent in our approach concerns the adherence of the participants to the research protocol, specifically the regular completion of the battery after each therapy session, and their compliance with the recommendation to share their emotional responses toward their therapist directly and openly with the therapist.

To this day, efforts to implement and test regular self-monitoring of patient affective experiences during sessions, with systematic feedback provided directly to patients without the therapist’s intervention, seem inadequate. The introduction of a newly developed systematic client feedback system for psychotherapy may offer improvements in treatment outcomes while also serving as a cost-effective tool in real-world clinical practice.

## Abbreviations

CORE-OM: Clinical Outcomes in Routine Evaluation-Outcome Measure
DSM-5: Diagnostic and Statistical Manual of Mental Disorders, Fifth Edition
GAD-7: Generalized Anxiety Disorder-7
PHQ-9: Patient Health Questionnaire-9
RISQ: Rift In-Session Questionnaire
RRI-C-SF: Real Relationship Inventory-Client-Short Form
SES: Session Evaluation Scale
SPARQ: in-Session Patient Affective Reactions Questionnaire
UNC: University of North Carolina
WAI-SR: Working Alliance Inventory-Short Revised

## Data Availability

Data sharing is not applicable to this article, as no data sets were generated or analyzed during this study protocol. The data sets generated during the actual research study will be made available in the Open Science Framework repository [31].

## References

[1] Ekman P, Dalgleish T, Power MJ (2005). Basic emotions. Handbook of Cognition and Emotion.

[2] Schore AN (2019). Right Brain Psychotherapy, 1st Edition.

[3] Stefana A (2015). The origins of the notion of countertransference. Psychoanal Rev.

[4] Stefana A (2017). History of Countertransference: From Freud to the British Object Relations School, 1st Edition.

[5] Stefana A (2017). Erotic transference. Brit J Psychotherapy.

[6] Chui H, Hill CE, Kline K, Kuo P, Mohr JJ (2016). Are you in the mood? Therapist affect and psychotherapy process. J Couns Psychol.

[7] Pascual-Leone A (2018). How clients "change emotion with emotion": a programme of research on emotional processing. Psychother Res.

[8] Faustino B (2022). Minding my brain: fourteen neuroscience-based principles to enhance psychotherapy responsiveness. Clin Psychol Psychother.

[9] Lane RD, Subic-Wrana C, Greenberg L, Yovel I (2022). The role of enhanced emotional awareness in promoting change across psychotherapy modalities. J Psychother Integr.

[10] Greenberg LS (2012). Emotions, the great captains of our lives: their role in the process of change in psychotherapy. Am Psychol.

[11] Schore AN (2012). The Science of the Art of Psychotherapy, 1st Edition.

[12] Greenberg LS, Goldman RN (2019). Theory of practice of emotion-focused therapy. Clinical Handbook of Emotion-Focused Therapy.

[13] Stefana A, Fusar-Poli P, Gnisci C, Vieta E, Youngstrom EA (2022). Clinicians' emotional reactions toward patients with depressive symptoms in mood disorders: a narrative scoping review of empirical research. Int J Environ Res Public Health.

[14] Stefana A, Youngstrom EA, Vieta E (2022). Empirical support for the use and further study of the countertransference construct in the clinical care of patients with bipolar disorder. Bipolar Disord.

[15] Greenberg LS, Greenberg LS, Goldman RN (2019). Theory of functioning in emotion-focused therapy. Clinical Handbook of Emotion-Focused Therapy.

[16] Subic-Wrana C, Greenberg LS, Lane RD, Michal M, Wiltink J, Beutel ME (2016). Affective change in psychodynamic psychotherapy: theoretical models and clinical approaches to changing emotions. Z Psychosom Med Psychother.

[17] Ulberg R, Hummelen B, Hersoug AG, Midgley N, Høglend PA, Dahl HSJ (2021). The First Experimental Study of Transference work-In Teenagers (FEST-IT): a multicentre, observer- and patient-blind, randomised controlled component study. BMC Psychiatry.

[18] Peluso PR, Freund RR, Norcross JC, Lambert MJ (2019). Emotional expression. Psychotherapy Relationships that Work. Volume 1: Evidence-Based Therapist Contributions, 3rd Edition.

[19] Hayes JA, Vinca M, Castonguay LG, Hill CE (2017). Therapist presence, absence, and extraordinary presence. How and why are Some Therapists Better than Others?: Understanding Therapist Effects.

[20] Barkham M, Broglia E, The SCORE Consortium (2023). Routine Outcome Monitoring (ROM) and feedback in university student counselling and mental health services: considerations for practitioners and service leads. Couns Psychother Res.

[21] Kendrick T, El-Gohary M, Stuart B, Gilbody S, Churchill R, Aiken L, Bhattacharya A, Gimson A, Brütt AL, de Jong K, Moore M (2016). Routine use of Patient Reported Outcome Measures (PROMs) for improving treatment of common mental health disorders in adults. Cochrane Database Syst Rev.

[22] de Jong K, Conijn JM, Gallagher RAV, Reshetnikova AS, Heij M, Lutz MC (2021). Using progress feedback to improve outcomes and reduce drop-out, treatment duration, and deterioration: a multilevel meta-analysis. Clin Psychol Rev.

[23] Østergård OK, Randa H, Hougaard E (2020). The effect of using the partners for change outcome management system as feedback tool in psychotherapy-a systematic review and meta-analysis. Psychother Res.

[24] Delgadillo J, Overend K, Lucock M, Groom M, Kirby N, McMillan D, Gilbody S, Lutz W, Rubel JA, de Jong K (2017). Improving the efficiency of psychological treatment using outcome feedback technology. Behav Res Ther.

[25] Delgadillo J, McMillan D, Gilbody S, de Jong K, Lucock M, Lutz W, Rubel J, Aguirre E, Ali S (2021). Cost-effectiveness of feedback-informed psychological treatment: evidence from the IAPT-FIT trial. Behav Res Ther.

[26] Janse PD, De Jong K, Van Dijk MK, Hutschemaekers GJM, Verbraak MJPM (2017). Improving the efficiency of cognitive-behavioural therapy by using formal client feedback. Psychother Res.

[27] Kazdin AE (2008). Evidence-based treatment and practice: new opportunities to bridge clinical research and practice, enhance the knowledge base, and improve patient care. Am Psychol.

[28] Norcross JC (2011). Psychotherapy Relationships that Work: Evidence-Based Responsiveness, 2nd Edition.

[29] Langkaas TF, Wampold BE, Hoffart A (2018). Five types of clinical difference to monitor in practice. Psychotherapy (Chic).

[30] Wampold BE (2015). How important are the common factors in psychotherapy? An update. World Psychiatry.

[31] Open Science Framework.

[32] Evans C, Connell J, Barkham M, Margison F, McGrath G, Mellor-Clark J, Audin K (2002). Towards a standardised brief outcome measure: psychometric properties and utility of the CORE-OM. Br J Psychiatry.

[33] Stefana A, Fusar-Poli P, Vieta E, Gelso CJ, Youngstrom EA (2024). Development and validation of an 8-item version of the Real Relationship Inventory-Client form. Psychother Res.

[34] Hatcher RL, Gillaspy JA (2006). Development and validation of a revised short version of the working alliance inventory. Psychother Res.

[35] Kroenke K, Spitzer RL, Williams JBW (2001). The PHQ-9: validity of a brief depression severity measure. J Gen Intern Med.

[36] Spitzer RL, Kroenke K, Williams JBW, Löwe B (2006). A brief measure for assessing generalized anxiety disorder: the GAD-7. Arch Intern Med.

[37] Stefana A, Langfus JA, Vieta E, Fusar-Poli P, Youngstrom EA (2023). Development and initial validation of the in-Session Patient Affective Reactions Questionnaire (SPARQ) and the Rift In-Session Questionnaire (RISQ). J Clin Med.

[38] Stefana A, Fusar-Poli P, Vieta E, Youngstrom E (2024). Assessing the patients’ affective perception of their psychotherapist: Validation of the in-Session Patient Affective Reactions Questionnaire (SPARQ). Front Psychiatry (forthcoming).

[39] Lent RW, Hoffman MA, Hill CE, Treistman D, Mount M, Singley D (2006). Client-specific counselor self-efficacy in novice counselors: relation to perceptions of session quality. J Couns Psychol.

[40] Harris PA, Scott KW, Lebo L, Hassan N, Lightner C, Pulley J (2012). ResearchMatch: a national registry to recruit volunteers for clinical research. Acad Med.

[41] Chandler J, Shapiro D (2016). Conducting clinical research using crowdsourced convenience samples. Annu Rev Clin Psychol.

[42] Guo S, Fraser MW (2010). Propensity Score Analysis: Statistical Methods and Applications.

[43] Goodman R (1999). The extended version of the strengths and difficulties questionnaire as a guide to child psychiatric caseness and consequent burden. J Child Psychol Psychiatry.

[44] Egbewale BE, Lewis M, Sim J (2014). Bias, precision and statistical power of analysis of covariance in the analysis of randomized trials with baseline imbalance: a simulation study. BMC Med Res Methodol.

[45] Twisk J, Proper K (2004). Evaluation of the results of a randomized controlled trial: how to define changes between baseline and follow-up. J Clin Epidemiol.

